# Laparoscopic Compared with Open D2 Gastrectomy on Perioperative and Long-Term, Stage-Stratified Oncological Outcomes for Gastric Cancer: A Propensity Score-Matched Analysis of the IMIGASTRIC Database

**DOI:** 10.3390/cancers13184526

**Published:** 2021-09-08

**Authors:** Stefano Trastulli, Jacopo Desiderio, Jian-Xian Lin, Daniel Reim, Chao-Hui Zheng, Felice Borghi, Fabio Cianchi, Enrique Norero, Ninh T. Nguyen, Feng Qi, Andrea Coratti, Maurizio Cesari, Francesca Bazzocchi, Orhan Alimoglu, Steven T. Brower, Graziano Pernazza, Simone D’Imporzano, Juan-Santiago Azagra, Yan-Bing Zhou, Shou-Gen Cao, Eleonora Garofoli, Claudia Mosillo, Francesco Guerra, Tong Liu, Giacomo Arcuri, Paulina González, Fabio Staderini, Alessandra Marano, Irene Terrenato, Vito D’Andrea, Sergio Bracarda, Chang-Ming Huang, Amilcare Parisi

**Affiliations:** 1 Department of Digestive Surgery, Azienda Ospedaliera Santa Maria, 05100 Terni, Italy; s.trastulli@aospterni.it (S.T.); a.parisi@aospterni.it (A.P.); 2 Department of Surgical Sciences—PhD Program in Advanced Surgical Technologies, Sapienza University of Rome, 00161 Rome, Italy; vito.dandrea@uniroma1.it; 3 Department of Gastric Surgery, Fujian Medical University Union Hospital, Fuzhou 350001, China; linjian379@fjmu.edu.cn (J.-X.L.); wwkzch@163.com (C.-H.Z.); hcmlr2002@163.com (C.-M.H.); 4 Klinik und Poliklinik für Chirurgie, Klinikum Rechts der Isar, Technische Universität München, 81675 Munich, Germany; daniel.reim@tum.de; 5 General and Oncologic Surgery Unit, Department of Surgery, Santa Croce e Carle Hospital, 12100 Cuneo, Italy; borghi.f@ospedale.cuneo.it (F.B.); marano.a@ospedale.cuneo.it (A.M.); 6 Digestive Surgery Unit, Department of Experimental and Clinical Medicine, “Careggi” Hospital, University of Florence, 50134 Florence, Italy; fabio.cianchi@unifi.it (F.C.); fabio.staderini@unifi.it (F.S.); 7 Esophagogastric Surgery Unit, Digestive Surgery Department, Hospital Dr. Sotero del Rio, Pontificia Universidad Catolica de Chile, Santiago 8207257, Chile; enorero@uc.cl (E.N.); pgonzac1@uc.cl (P.G.); 8 Irvine Medical Center, Department of Surgery, Division of Gastrointestinal Surgery, University of California, Orange, CA 92868, USA; ninhn@uci.edu; 9 Gastrointestinal Surgery, Tianjin Medical University General Hospital, Tianjin 300052, China; qf@medmail.com.cn (F.Q.); liutonga@126.com (T.L.); 10 Department of General and Emergency Surgery, Division of General and Emergency Surgery, School of Robotic Surgery, Misericordia Hospital of Grosseto, 58100 Grosseto, Italy; andrea.coratti@uslsudest.toscana.it (A.C.); francesco.guerra@uslsudest.toscana.it (F.G.); 11 Department of General Surgery, Hospital of Città di Castello, USL1 Umbria, 06012 Città di Castello, Italy; cesarimaurizio@tiscali.it; 12 Department of Surgery, Fondazione IRCCS Casa Sollievo della Sofferenza, 71013 San Giovanni Rotondo, Italy; f.bazzocchi@operapadrepio.it; 13 Department of General Surgery, School of Medicine, Istanbul Medeniyet University, 34000 Istanbul, Turkey; orhan.alimoglu@medeniyet.edu.tr; 14 Department of Surgical Oncology and HPB Surgery, Englewood Hospital and Medical Center, Englewood, NJ 07631, USA; steven.brower@ehmchealth.org; 15 Robotic General Surgery Unit, Department of Surgery, San Giovanni Addolorata Hospital, 00184 Rome, Italy; gpernazza@hsangiovanni.roma.it; 16 Esophageal Surgery Unit, Tuscany Regional Referral Center for the Diagnosis and Treatment of Esophageal Disease, Medical University of Pisa, 56124 Pisa, Italy; simone.dimporzano@alice.it; 17 Unité des Maladies de l’Appareil Digestif et Endocrine, Centre Hospitalier de Luxembourg, 1210 Luxembourg, Luxembourg; azagra.js@chl.lu; 18 Department of General Surgery, The Affiliated Hospital of Qingdao University, Qingdao 266003, China; zhouyanbing999@aliyun.com (Y.-B.Z.); briancao@126.com (S.-G.C.); 19 Medical and Translational Oncology, Department of Oncology, Azienda Ospedaliera Santa Maria, 05100 Terni, Italy; e.garofoli@aospterni.it (E.G.); c.mosillo@aospterni.it (C.M.); s.bracarda@aospterni.it (S.B.); 20 Division of Surgery, S. Maria della Misericordia Hospital, 06129 Perugia, Italy; giacomo_arcuri@alice.it; 21 Biostatistics and Bioinformatic Unit, Scientific Direction, IRCCS Regina Elena National Cancer Institute, 00128 Rome, Italy; ireneter@gmail.com

**Keywords:** gastric cancer, laparoscopy, minimally invasive surgery

## Abstract

**Simple Summary:**

Gastric resection with D2 lymphadenectomy is considered the gold standard for the treatment of both advanced and early gastric cancer with lymph node metastasis. The performance of D2 lymphadenectomy is technically challenging and represents a key factor in improving patients’ survival. For these reasons, the execution of gastrectomy with D2 lymphadenectomy using the traditional open surgical technique still represents the most widespread approach and, based on current international guidelines, the indication for laparoscopic surgery is limited to early gastric cancer that does not require a D2 lymphadenectomy. The present study aimed to investigate the use of laparoscopic versus open surgical approaches in performing gastrectomy with D2 lymphadenectomy for cancer in terms of intraoperative and postoperative outcomes and long-term survival. The study was conducted using the data collected in the International study group on Minimally Invasive surgery for Gastric Cancer (IMIGASTRIC) international database.

**Abstract:**

Background: The laparoscopic approach in gastric cancer surgery is being increasingly adopted worldwide. However, studies focusing specifically on laparoscopic gastrectomy with D2 lymphadenectomy are still lacking in the literature. This retrospective study aimed to compare the short-term and long-term outcomes of laparoscopic versus open gastrectomy with D2 lymphadenectomy for gastric cancer. Methods: The protocol-based, international IMIGASTRIC (International study group on Minimally Invasive surgery for Gastric Cancer) registry was queried to retrieve data on patients undergoing laparoscopic or open gastrectomy with D2 lymphadenectomy for gastric cancer with curative intent from January 2000 to December 2014. Eleven predefined, demographical, clinical, and pathological variables were used to conduct a 1:1 propensity score matching (PSM) analysis to investigate intraoperative and recovery outcomes, complications, pathological findings, and survival data between the two groups. Predictive factors of long-term survival were also assessed. Results: A total of 3033 patients from 14 participating institutions were selected from the IMIGASTRIC database. After 1:1 PSM, a total of 1248 patients, 624 in the laparoscopic group and 624 in the open group, were matched and included in the final analysis. The total operative time (median 180 versus 240 min, *p* < 0.0001) and the length of the postoperative hospital stay (median 10 versus 14.8 days, *p* < 0.0001) were longer in the open group than in the laparoscopic group. The conversion to open rate was 1.9%. The proportion of patients with in-hospital complications was higher in the open group (21.3% versus 15.1%, *p* = 0.004). The median number of harvested lymph nodes was higher in the laparoscopic approach (median 32 versus 28, *p* < 0.0001), and the proportion of positive resection margins was higher (*p* = 0.021) in the open group (5.9%) than in the laparoscopic group (3.2%). There was no significant difference between the groups in five-year overall survival rates (77.4% laparoscopic versus 75.2% open, *p* = 0.229). Conclusion: The adoption of the laparoscopic approach for gastric resection with D2 lymphadenectomy shortened the length of hospital stay and reduced postoperative complications with respect to the open approach. The five-year overall survival rate after laparoscopy was comparable to that for patients who underwent open D2 resection. The types of surgical approaches are not independent predictive factors for five-year overall survival.

## 1. Introduction

Gastric cancer is the fifth most common malignancy worldwide and the third leading cause of cancer death globally [1].

Gastrectomy with adequate lymphadenectomy still represents the treatment of choice to obtain radical resection and achieve better survival outcomes in the case of resectable gastric cancer.

Based on the current international guidelines, D2 lymphadenectomy should be performed in the event of T2-4aN0-3M0 stage gastric cancer [2].

The laparoscopic approach in gastric cancer surgery was first described in 1994 to perform distal gastrectomy for early gastric cancer [3].

The current Japanese guidelines for gastric cancer treatments consider laparoscopic surgery as an option to treat cStage I cancer that is resectable with a distal gastrectomy and indicate that, for advanced gastric cancer, some concern still exists based on the available evidence in the literature, mainly concerning the survival endpoints with respect to the open approach, particularly for total gastrectomy [2].

However, primarily thanks to the well-established benefits of laparoscopy in early postoperative outcomes, the laparoscopic approach is also being increasingly adopted for the performance of gastrectomy with D2 lymphadenectomy [4].

Randomized controlled trials (RCTs) represent the gold standard of medical evidence for assessing the efficacy and safety of therapeutic interventions. To date, many published RCTs [5,6,7,8,9,10,11,12] have specifically investigated the laparoscopic versus open D2 lymphadenectomy, but only two of these studies reported data on five-year survival; yet, these studies included only distal gastrectomies [7] or a small proportion of total gastrectomies [10]. The other RCTs on laparoscopic D2 lymphadenectomy, which did not assess five-year survival data, focused only on distal gastrectomy [6,12] or included only a small or very small proportion of total gastrectomy procedures in their analysis [5,8,9,11].

Observational studies based on large databases may notably represent an acceptable methodological alternative to RCTs if propensity score matching is applied to reduce inference biases and distortions potentially introduced by uncontrolled confounders. Some observational large database studies have already been conducted to investigate laparoscopic D2 lymphadenectomy with respect to the open approach [13,14,15,16,17], but these studies were all single-institution studies conducted in eastern countries. Some of these studies also included a proportion of D1 lymphadenectomy procedures and/or a smaller proportion of total gastrectomies or did not perform a case-matched analysis.

Based on this background, the present study aimed to investigate the short-term intraoperative surgical and pathological endpoints as well as postoperative recovery and long-term (five-year) survival outcomes of laparoscopic total and distal gastrectomy with D2 lymphadenectomy for gastric cancer in comparison to the open approach by analyzing data from a large international database (western and eastern centers), the International study group on Minimally Invasive surgery for Gastric Cancer (IMIGASTRIC) database, with the adoption of propensity score matching analysis.

## 2. Materials and Methods

### 2.1. Overall Objective and Type of Study

The objective of this retrospective case-matched study was to compare laparoscopic versus open gastrectomy with D2 lymphadenectomy in terms of clinical, surgical, and long-term oncological outcomes using the retrospective data stored in the IMIGASTRIC database (www.imigastric.com (accessed on 20 December 2020)). IMIGASTRIC is a protocol-based, international, multi-institutional registry aimed at retrieving clinical, surgical, and oncological variables of patients undergoing laparoscopic, robotic, or open surgery for gastric cancer [18]. The retrospective data extracted for the present study were shared by a total of 14 institutions worldwide (Chile, China, Germany, Italy, Luxembourg, Turkey, and the USA) that participated in the IMIGASTRIC registry at the time the data extraction was performed (January 2018). The surgical procedures were carried out in the participating institutions from January 2000 to August 2018. During this period, all institutions treated patients with gastric cancer according to international guidelines. The present study was conducted after approval by the institutional review board of each participating institution.

### 2.2. Inclusion and Exclusion Criteria

Eligible patients were identified from the IMIGASTRIC database based on the following inclusion and exclusion criteria:

Inclusion criteria: (1) histologically proven gastric adenocarcinoma, (2) preoperative staging workup performed by upper endoscopy and/or endoscopic ultrasound, and CT scan, (3) total or distal gastrectomy with D2 lymphadenectomy using the laparoscopic or open surgical approach, (4) treatment with a curative intent in accordance with international guidelines, (5) availability of follow-up data (alive, died, or lost to follow-up), (6) surgery performed in the participating institutions from January 2000 to December 2014.

Exclusion criteria: (1) evidence of metastatic tumor (peritoneal carcinomatosis, liver metastasis, distant lymph node metastasis, Krukenberg tumors, involvement of other organs), (2) American Society of Anesthesiologists (ASA) score ≥4, (3) history of previous abdominal surgery for gastric cancer, (4) synchronous malignancy in other organs, (5) palliative surgery, (6) in situ neoplasms.

### 2.3. Data Collection and Outcomes

The following demographic, clinical, and pathological variables were recorded for each selected patient: age, sex, body mass index (BMI, kg/m^2^), ASA score, comorbidities (present/absent), geographic area (east/west), type of gastric resection (total or distal gastrectomy), year of surgery, tumor location (upper, middle, or distal third of the stomach), tumor histology based on the Japanese classification of gastric carcinoma, 3rd English edition [19] (differentiated, poorly differentiated/undifferentiated, and subtypes tubular well-differentiated, tubular moderately differentiated, papillary, mucinous, signet ring cell, poorly differentiated solid/non-solid type, undifferentiated), and tumor pathological TNM (pTNM) stage. Cancer stage was determined according to the AJCC/UICC TNM staging system, 8th edition [20].

The following intraoperative and postoperative findings were considered as the study endpoints: total operative time (minutes), conversion to open surgery rate, number of harvested lymph nodes, R factors (R0, R1, R2), number of metastatic lymph nodes, detailed histological types (tubular well-differentiated, tubular moderately differentiated, papillary, mucinous, signet ring cell, poorly differentiated solid/non-solid type, undifferentiated), postoperative hospital stay (days), number of patients with in-hospital complications, grade of in-hospital complications according to Clavien-Dindo classification [21], the proportion of patients undergoing neoadjuvant chemo or radiotherapy, proportion of in-hospital complications with a severity of grade 3 or more according to the Clavien-Dindo classification (severe complications), number of each in-hospital complication, reoperation for complications, and in-hospital mortality rates. The five-year overall survival was investigated, also stratifying patients according to the AJCC/UICC TNM staging system, 8th edition [20], and regression analysis was carried out to identify relevant predictors for overall survival.

### 2.4. Statistical Analysis

Categorical variables were summarized using frequencies and percentage values, while continuous variables were summarized using mean values and their relative standard deviation (SD) and median with the relative interquartile range (IQR).

To control for potential confounders, propensity score matching (PSM) was performed to create two treatment groups with a balanced distribution of baseline demographic, clinical, and pathological features. A total of 11 variables were used in a logistic regression model to calculate the propensity score: age, sex, BMI, ASA score, year of surgery (2000–2007/2008–2014), comorbidities (present/absent), geographic area (east/west), type of gastric resection (total or distal gastrectomy), tumor location (upper, middle, or distal third), tumor histology (differentiated or poorly differentiated/undifferentiated), and tumor pathological TNM (pTNM) stage. The proportion of patients who underwent laparoscopic or open surgery in each participating institution was highly variable, and the use of PSM for this specific parameter was considered to be difficult. Patients were matched with a 1:1 ratio using the nearest-neighbor method without replacement and with a caliper of 0.05 of the SD of the logit of the estimated propensity score. Patients who were found to be outside of the caliper were excluded from the analysis. After matching, the balance within groups was evaluated using the overall balance test by Hansen and Bowers and the relative multivariate imbalance measure, L1, proposed by Iacus, King, and Porro.

The chi-square test or Fisher’s exact test, when appropriate, was used to compare categorical variables, and the student’s *t*-test or the Mann–Whitney non-parametric U test was applied to compare continuous variables.

Overall survival was calculated using the Kaplan–Meier method, and the log-rank test (Mantel–Hanszel) was used to assess differences in five-year overall survival between study groups. Survival analyses were performed for the entire study cohort and for the propensity score-matched cohorts to adjust for significant differences in the clinical and pathological characteristics. Overall survival was calculated from the date of surgery until the date of death or until the date of the last contact. The follow-up and the survival time were reported as the median and interquartile range (IQR, 25–75° percentiles).

To investigate potential predictors of overall survival, the Hazard ratio and its relative 95% confidence interval (95% CI) were calculated using the Cox proportional univariate regression model. Then, a multivariate Cox proportional hazard model was generated using stepwise regression (forward selection with the entry limit of *p* = 0.05 and the remove limit of *p* = 0.10).

All analyses were two-tailed, and *p*-values < 0.05 were considered to be statistically significant. Additionally, all analyses were conducted with the “intention to treat” method. All statistical analyses were performed using SPSS software version 23.0 (SPSS Inc., Chicago, IL, USA) and GraphPad software version 6.01 (La Jolla, CA, USA).

## 3. Results

### 3.1. Patient Selection

**Figure 1:**
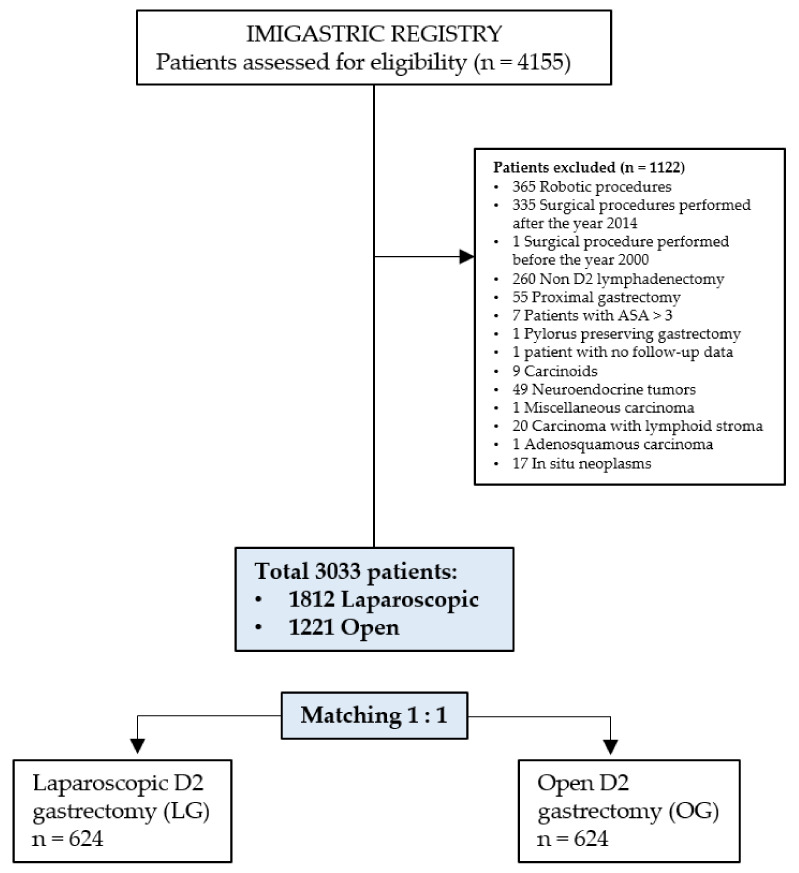
Study flow-chart.

As indicated in Figure 1, a total of 4155 patients affected by gastric adenocarcinoma and registered in the IMIGASTRIC registry underwent total or distal gastrectomy and were therefore considered for inclusion in the study. After the application of the predefined inclusion and exclusion criteria, a total of 1122 patients were excluded (reasons for exclusion are indicated in Figure 1). Finally, a total of 3033 patients were included in the study, of whom 1812 underwent laparoscopic D2 gastric resection and 1221 underwent open D2 gastric resection for gastric adenocarcinoma. After 1:1 matching using the propensity score, two well-balanced groups, one composed of 624 patients undergoing laparoscopic D2 gastrectomy and one 624 patients undergoing open D2 gastrectomy, were selected for data analysis.

### 3.2. Patient Characteristics

Considering the entire patient cohort before the matching procedure and comparing the two study groups, a statistically significant difference was found in the distribution of gender, geographic area, year of surgery, ASA score, comorbidities, tumor location, pTNM stage, and histology, and in terms of average BMI between the laparoscopic and open approach groups (Supplementary Table S1). After statistical matching, the two study groups were found to be well balanced for all considered clinical, surgical, and demographic variables, as indicated in Table 1.

### 3.3. Operative Outcomes

The operating findings, postoperative complications, and pathology data analyzed comparing the two study groups and considering both the matched cohort and the entire patient cohort are summarized in Table 2 and in Supplementary Table S2, respectively. In this section, only the results of the comparison of the two study groups for the matched cohort of patients are reported.

In the matched cohort, the total operative time was significantly longer in the open group than in the laparoscopic group (median 180 versus 240 min, *p* < 0.0001). The laparoscopic to open conversion rate was 1.9% (total of 12 procedures converted to open surgery).

The median number of harvested lymph nodes was significantly higher in the laparoscopic group than in the open group (median 32 versus 28 harvested lymph nodes, *p* < 0.0001) but the median number of metastatic lymph nodes was similar in the two groups (median 3 metastatic lymph nodes in both study groups, *p* = 0.400).

The proportion of positive resections (R1 or R2) was significantly higher (*p* = 0.021) in the open group (5.9%) than in the laparoscopic group (3.2%).

No significant differences in terms of the proportion of patients undergoing neoadjuvant chemotherapy (*p* = 0.493) or neoadjuvant radiotherapy (*p* = 1.000) were found comparing the two study groups.

### 3.4. Histology

The distribution of the tumor histology types between the two study groups was similar (*p* = 0.767). In both study groups, the tubular well-differentiated type represented the most common histotype among the differentiated tumors, and the signet ring cell histotype was the most common histotype among the poorly differentiated/undifferentiated tumors.

### 3.5. Post-Operative Outcomes

In the laparoscopic group, a significantly shorter length of postoperative hospital stay was found than in the open group (median 10 versus 14.8 days, *p* < 0.0001).

The proportion of patients with at least one in-hospital complication was significantly higher in the open group than in the laparoscopic group (21.3% versus 15.1%, *p* = 0.004). The distribution of in-hospital complications classified with the Clavien–Dindo score was similar between the laparoscopic and open groups (*p* = 0.433), as was the proportion of severe (Clavien–Dindo grade ≥3) in-hospital complications (19% versus 20.8%, *p* = 0.686).

No significant differences were found when comparing the laparoscopic and open approaches in terms of the proportion of reoperation rates for in-hospital complications (1.6% versus 2.7%, *p* = 0.392) and in terms of in-hospital mortality rates (0.2% versus 0.3%, *p* = 0.563).

In Table 3, the number of in-hospital complications are reported in detail for the matched study cohort. In the matched cohort, a statistically significant difference was found in the proportion of postoperative in-hospital complications between the laparoscopic and open surgical approaches only in terms of pneumonia (*p* = 0.003), which was higher in the open group (9.8%) than in the laparoscopic group (5.3%). No significant differences were found for the remaining postoperative complications when comparing the two study groups.

### 3.6. Survival Analysis

In Table 4, the results of the survival analysis in terms of five-year overall survival rates using the Kaplan–Meier method in both the entire and matched study cohort are reported.

The follow-up duration in the entire study cohort was a median of 62 (IQR 38–78) months and a median of 60 (IQR 35–75) months in the matched study cohort. In the laparoscopic matched cohort, the median follow-up time was 63 (IQR 44–77) months, whereas in the open group the median follow-up time was 57 (IQR 24–72) months. The proportion of patients lost to follow-up was 9.2% and 14.1% in the entire and in the matched cohort, respectively. In the matched cohort, the median observation time in the population lost to follow-up was 21 (IQR 12–30) months in the laparoscopic group and 14 (IQR 2–57) months in the open group.

**Figure 2:**
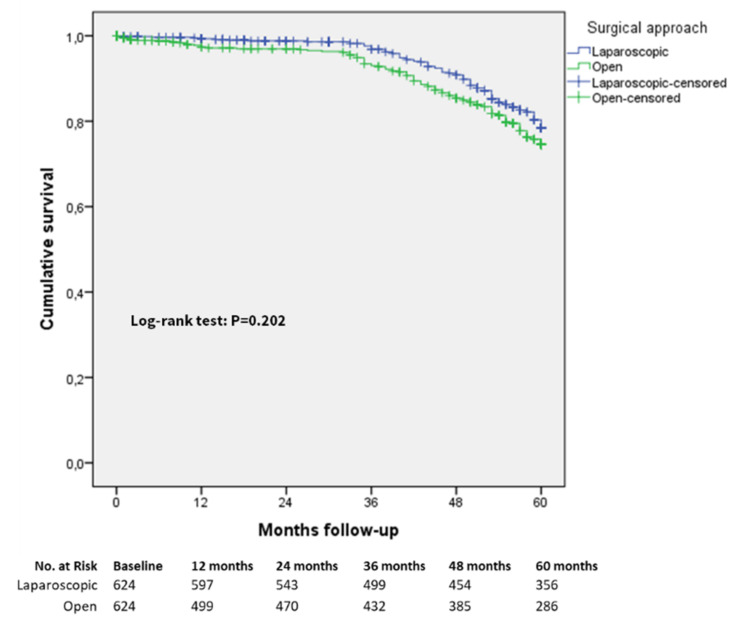
Five-year overall survival, matched cohort (all patients).

**Figure 3:**
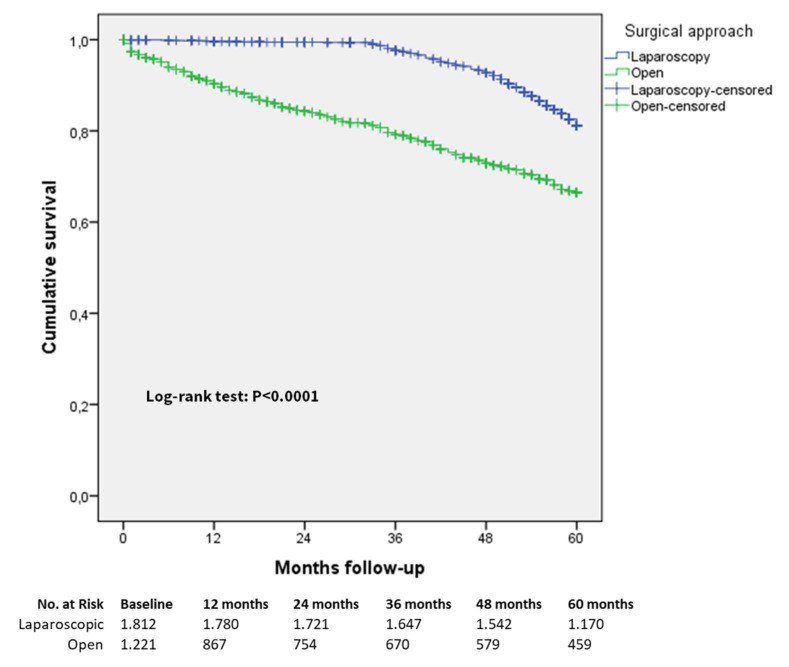
Five-years overall survival, entire cohort (all patients).

The comparison of five-year overall survival rates in the laparoscopic and open groups considering all included patients irrespective of cancer staging in the matched group did not reach a statistically significant difference, as indicated in Figure 2 (rate of 77.4% versus 75.2%, respectively, log-rank (Mantel–Cox) test *p* = 0.202) with a mean survival time in the laparoscopic group of 79.26 (95% CI 76.90 to 81.62) months versus 81.99 (95% CI 76.62 to 87.36) months in the open group. A total of 493 patients died during the follow-up in the matched cohort, of whom 251 were in the laparoscopic group and 242 were in the open group. In Figure 3, the comparison of five-year overall survival rates between the laparoscopic and open groups in the entire study cohort are shown, with a significant survival advantage for the laparoscopic group (rate of 81.1% versus 66.4%, log-rank (Mantel–Cox) test *p* < 0.0001).

**Figure 4:**
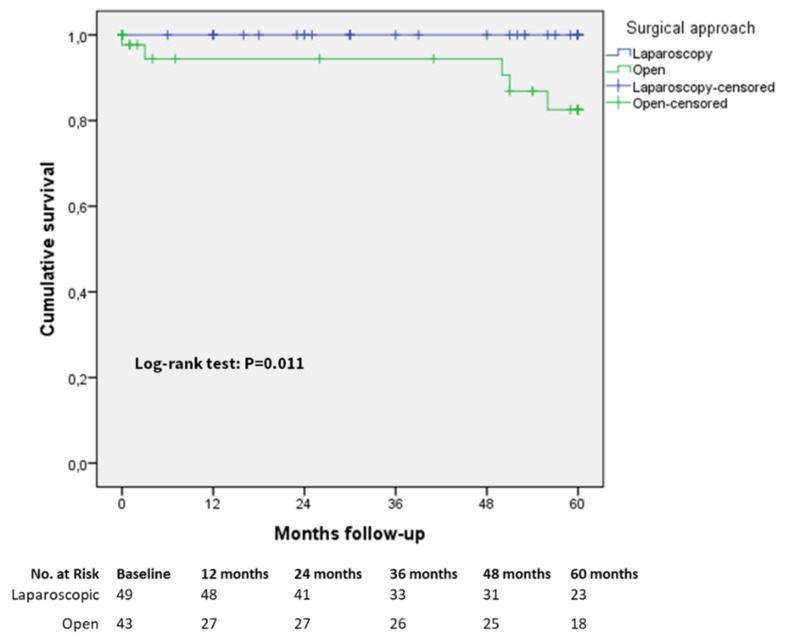
Five-year overall survival matched cohort in stage IB patients.

The five-year overall survival rates did not significantly differ in the matched group between the laparoscopic and open approaches, also considering separately each cancer stage as indicated in Table 4 and in Supplementary Figures S1–S9, except for the patients with cancer stage IB (Figure 4), for which five-year survival rates were 100% versus 82% in the laparoscopic and open groups, respectively (Log-rank (Mantel–Cox) test *p* = 0.011).

As indicated in Table 5, the univariate analysis showed that the age, gender, geographic area, year of surgery, BMI, ASA score, operative time, type of surgical approach, length of hospital stay, type of resection, tumor location, number of harvested lymph nodes, margin status, TNM stage, number of metastatic lymph nodes, and histology were significantly associated with overall survival. The stepwise multivariable Cox regression analysis showed that the following variables were predictive factors for overall survival: age (HR 1.019, 95% CI 1.011 to 1.027), geographic area considering the “east” geographic area as a reference parameter (west, HR 6.482, 95% CI 5.322–7.895), ASA score, considering as a reference parameter ASA score I (ASA score II, HR 1.273, 95% CI 1.065–1.521), tumor location, considering as a reference parameter the proximal third (distal third, HR 0.772, 95% CI 0.633–0.941), TNM stage, considering as a reference parameter stage IIIC (stage IA, HR 0.057, 95% CI 0.032–0.102; stage IB, HR 0.171, 95% CI 0.102–0.288, stage IIA, HR 0.261, 95% CI 0.165–0.412; stage IIB, HR 0.282, 95% CI 0.17–0.443; stage IIIA, HR 0.645 95% CI 0.454–0.916), and the number of metastatic lymph nodes (HR 1.018 95% CI 1.006–1.030).

## 4. Discussion

### 4.1. Study Findings (Short-Term Outcomes)

The present multicenter, registry-based study enrolled a total of 3033 patients representing, to date, the larger comparative study investigating laparoscopic versus open D2 gastrectomy for cancer.

By adopting the case-matched study design, we were able to compare clinical, pathological, and survival outcomes in a total of 1248 patients, 624 in each study group, who were selected from the IMIGASTRIC registry according to 11 predefined covariates to control for possible confounders.

Our study indicates that the laparoscopic approach for total or distal gastrectomy with D2 lymphadenectomy is capable of significantly reducing, with respect to the open approach, the total operating time (median 180 versus 240 min respectively, *p* < 0.0001), the length of postoperative hospital stay (median 10 versus 14.8 days, respectively, *p* < 0.0001) as well as the number of postoperative in-hospital complications (142 versus 173, respectively, *p* = 0.043) and the proportion of patients with at least one in-hospital complication (15.1% versus 21.3%, respectively, *p* = 0.004), despite the occurrence of postoperative severe complications, based on the Clavien–Dindo classification, with results similar between the open and laparoscopic approaches (19% versus 20.8%, respectively, *p* = 0.686).

No significant differences were found in terms of in-hospital mortality and reoperation rates. The rates of in-hospital mortality found in our analysis were quite low if compared to those reported in other large multicenter studies on gastric cancer surgery, such as the German Gastric Cancer Study 2 (QCGC 2) [22], in which the hospital mortality rate was overall 5.8% in the included high surgical volume centers. We can explain this wide variation by the fact that a large proportion of patients included in our analysis were treated in selected, very high volume centers dedicated to gastric cancer treatment that are mainly located in the east, particularly in China. The in-hospital mortality reported by the eastern high volume gastric cancer centers is notably very low as confirmed by the findings of a recent Cochrane review on randomized controlled trials, mainly conducted in the east, indicating an overall short-term mortality rate of about 0.5% after laparoscopic or open gastrectomy for cancer [23].

### 4.2. Operative Time

We can argue that the findings of a significantly shorter operative time in the laparoscopic group than in the open group in the present study could be linked with a selection bias derived from the inclusion of less technically demanding cases in the laparoscopic group despite the adoption of a case matching analysis that reduced the influence of some confounders.

Moreover, it should be underlined that in the literature, some other studies investigating open versus laparoscopic gastrectomy for advanced cancer have indicated a shorter operative time in favor of the laparoscopic approach [24,25,26], while other studies have shown no differences between these approaches in terms of operative time [17,27], confirming that the completion of an appropriate learning curve allows for a significant reduction in the operation time in the laparoscopic approach up to matching or overcoming that required for the open approach [28].

### 4.3. Hospital Stay and Complications

A recent systematic review with a meta-analysis on laparoscopic versus open gastrectomy for advanced cancer confirmed a significant reduction in the length of hospital stay after the laparoscopic approach with a reduction in the postoperative length of stay of about three days [29], which was comparable with our findings. The reduction in the length of hospital stay represents a notable and well-known advantage of laparoscopic surgery and is mainly linked with the minimal gastrointestinal stress and minimal abdominal incision offered by laparoscopy.

Our analysis showed a significant reduction of postoperative in-hospital complications for patients undergoing laparoscopy both in terms of the overall complication rate and the proportion of patients with a least one complication (15.1%); this is in the range of postoperative morbidity reported in the literature focusing on the laparoscopic treatment of advanced gastric cancer, which ranged between 6.3% and 24.2% [12,16,30,31,32,33,34]. By specifically analyzing the proportion of each reported postoperative complication, we found that only the occurrence of pneumonia was significantly higher in the open group. The reduction of pulmonary complications, including pneumonia, is a well-known advantage related to the adoption of laparoscopy, not only in gastric surgery [35].

### 4.4. Lymphadenectomy

The AJCC 8th TNM-staging guidelines [20] indicate a minimum of 16 lymph nodes to be assessed in radical gastric cancer surgery, with 30 lymph nodes being desirable. Some studies have demonstrated a survival benefit linked with the increased number of harvested lymph nodes, with the maximal survival advantage reached with the dissection of 29 nodes [36,37]. Although the majority of studies comparing gastrectomy with laparoscopic versus open D2 lymphadenectomy found no significant difference in the number of retrieved lymph nodes [4], some studies have indicated significantly more harvested lymph nodes with laparoscopy [24,27,32,38,39,40]. Also in the present study, we found that patients in the laparoscopic group had a significantly higher median number of harvested lymph nodes than in the open group (median of 32 versus 28 harvested lymph nodes, *p* < 0.0001). This finding could be partly explained by the technical advantages offered by laparoscopy in terms of a magnified view of the surgical field, allowing for finer and deeper tissue dissection, in turn facilitating a more extensive lymphadenectomy than open surgery, in adequately experienced hands [41]. The same technical advantages of laparoscopy could explain the findings of a significantly higher *p* = 0.021) proportion of positive surgical resection margins in the open (5.9%) versus laparoscopic (3.2%) approaches in the present analysis.

### 4.5. Long-Term Survival and Related Factors

As previously underlined, only two RCTs on laparoscopic versus open D2 lymphadenectomy reported data on five-year survival, but these studies included only distal gastrectomies [7] or a small proportion of total gastrectomies [10]. The five-year overall survival rates calculated in our study for each cancer stage in the laparoscopic (stage I: 96.4%, stage II: 86.7%, stage III: 67.8%) and open (stage I: 94%, stage II: 83.8%, stage III: 65%) groups, which included well-balanced groups of total and distal gastrectomies, are in line with the survival rate reported in previous observational studies [31,42,43]. In our analysis, only the five-year overall survival rate in the stage IIB patients was significantly higher in the laparoscopic group (100%) than in the open group (82%). However, this finding is probably linked with a type 2 statistical error for this specific subgroup of patients due to the small sample size of the stage IIB patients in the laparoscopic (*n* = 49) and open (*n* = 43) groups in the matched study cohort. Overall, our data confirm that the adoption of the laparoscopic approach in gastric cancer resection with D2 lymphadenectomy is capable of providing pathological and long-term survival endpoints comparable to those of the open approach.

This evidence is also supported by the findings of the present study indicating that the surgical approach (laparoscopic or open) was not an independent prognostic factor for five-year overall survival after multivariable regression analysis. Conversely, age, geographic area (west versus east), ASA score, tumor location (distal), pTNM stage, and the number of metastatic lymph nodes were identified as independent prognostic factors for five-year overall survival after univariate and multivariate analysis of the entire cohort of patients. Interestingly, our multivariable regression analysis showed the five-year risk of death increased 6.4 times more in patients treated in the west geographic area than in the east area. A recent survival analysis conducted in the German Gastric Cancer Study 2 (QCGC 2) [22] showed a five-year overall survival rate of 38.7%, calculated on the included high surgical volume centers, which is about half of that obtained in the present study, which included a large proportion of patients treated in very high volume eastern centers. Although the observed differences in five-year survival between eastern and western cohorts can be largely explained by differences in baseline demographic, clinical, and pathological characteristics [44], some studies have indicated the number of lymph nodes examined to be a key contributing factor to the observed survival difference, which was also related to the well-known phenomenon of “stage migration” [45,46]. On the other hand, the number of examined lymph nodes is dependent on some factors, such as the extent of the surgery, the examiner’s technique, fat volume in the specimen, or the innate number of the lymph nodes. Some of these factors, such as the extent of surgery and the pathological examiner’s technique, are correlated with the institution’s surgical case volume and to the consequent surgical expertise that is, in general, greater in eastern centers due to both the higher incidence of gastric cancer and the earlier and longer adoption of the surgical principles of oncological radicality, especially inherent to D2 lymphadenectomy, in eastern rather than in western countries.

In the present study, the multivariable analysis showed that the location of the tumor in the distal portion of the stomach was linked with an improved five-year overall survival of about 23% with respect to the proximal tumor location despite the performance of distal versus total gastrectomy not being an independent predictive factor for overall survival. Similar to our findings, a recent systematic review investigating the prognostic role of primary tumor location in non-metastatic gastric cancer [47] found a 25% increased risk of mortality of proximal compared with distal gastric cancers assuming that this evidence is related to more aggressive biological characteristics of the proximal tumors.

### 4.6. Strengths and Limitations of the Study

Although this registry-based analysis represents, to date, the study with the largest sample size investigating laparoscopic versus open D2 lymphadenectomy for the treatment of gastric cancer, including a well-represented end-balanced subgroup of patients undergoing total and distal gastrectomy, it has some limitations. First of all, this was a retrospective study and, despite the adoption of the case matching analysis, the risk of unmeasurable bias in the analysis persisted, especially of selection bias in favor of the laparoscopic approach. The patient study cohort was retrieved from the IMIGASTRIC database, which is an international, multi-institutional registry with participating centers located in the east and west geographic areas of the world, with the risk of substantial general heterogeneity in some aspects of gastric cancer treatments. Despite the analyzed data coming from centers located on three different continents (Europe, America, Asia), it should be acknowledged that most of the analyzed surgical procedures were performed in eastern countries and in particular in China; therefore, the results cannot be extrapolated to all gastric cancer patients, in particular from western countries.

It should also be pointed out that the IMIGASTRIC database was established and is maintained based on a dedicated protocol [18] regulating the collection, management, and analysis of data that was shared, between the participating centers, at the beginning of the data collection. The definitions of the clinical, pathological, and surgical parameters selected for storage in the IMIGASTRIC database are specified in the study protocol, allowing for a reduction in the heterogeneity and inconsistency of data analysis. These aspects markedly differentiate the IMIGASTRIC database from the databases created to store administrative data, which are to date increasingly used in clinical research. Studies conducted on these large administrative databases, which are mostly based in the USA (such as the Nationwide Inpatient Sample (NIS) and Medicare database) are burdened by the peculiar bias defined as “coding bias” [48,49].

The assessment of disease-free survival and disease recurrence and patterns would certainly increase the completeness of the survival analysis in the present study. Unfortunately, the data needed to perform these specific survival analyses are not yet available in the IMIGASTRIC registry. However, it should be noted that to date only two RCTs have reported data on five-year survival, but these RCTs included only distal gastrectomy [7] or a small proportion of total gastrectomies [10], and observational studies analyzing five-year overall survival assessed samples of patient smaller than that in our study and/or did not adopt the case-matched design or included only small subgroups of patients undergoing total gastrectomy [14,15,16,41,50].

The patients enrolled in the present study were treated over a rather long time, from January 2000 until December 2014. This could theoretically pose the risk of patients treated in different historical periods having been subjected to different surgical and neoadjuvant/adjuvant treatments. This choice was based on the necessity to collect a sample of patients as large as possible to conduct analysis with satisfactory statistical power and based on the evidence that the guidelines on the surgical and chemo-radiotherapy treatment of gastric cancer, in particular regarding the procedures for performing D2 lymphadenectomy, were published in 1998 [51].

Moreover, the adjuvant chemotherapy treatments did not present, in the time frame identified for the collection of these retrospective data, substantial changes in terms of indications, types of drugs, and the chemo-radiotherapy schemes used. Furthermore, adjuvant treatments (chemo and radiotherapy) for advanced gastric cancer showed only a modest effect in improving the survival of patients (about 7 months in a meta-analysis of 64 randomized studies) [52]. Even the efficacy and indications for the use of neoadjuvant (preoperative) chemotherapy treatment remains highly controversial and is therefore not routinely performed worldwide [53].

## 5. Conclusions

Our study, despite the aforementioned limits, mainly related to its retrospective nature, suggests that laparoscopic gastric resection with D2 lymphadenectomy for gastric cancer allows the retrieval of better recovery outcomes with respect to the open approach by reducing the hospital stay and the postoperative overall morbidity with no significant differences in terms of short-term pathological outcomes and long-term survival, after both total and distal gastrectomy. The findings of the present study, which contribute to enriching the existing literature on minimally invasive D2 lymphadenectomy, could also be useful for planning further RCTs, which are still strongly needed to definitively assess the benefits and possible harms of laparoscopic gastrectomy with D2 lymphadenectomy with respect to the open approach, particularly in western countries.

## Figures and Tables

**Table 1:** Clinical, surgical, and demographic patient characteristics by surgical technique compared in the matched cohort.

	Matched Cohort		
	Laparoscopic *n* = 624	Open *n* = 624	*p*-Value
**Age, years**			0.985 ^#^
median (IQR)	63 (6–72)	64 (56–72)	
mean (SD)	63.2 (11.3)	63 (11.9)	
**Gender, *n* (%)**			0.849
Male	466 (73.1)	453 (72.6)	
Female	168 (26.9)	171 (27.4)	
**Geographic area, *n* (%)**			0.829
East	504 (80.8)	507 (81.3)	
West	120 (19.2)	117 (18.8)	
**Year of surgery, *n* (%)**			1.000
2000–2007	16 (2.6)	16 (2.6)	
2008–2014	608 (97.4)	608 (97.4)	
**Body Mass Index (BMI)**			0.350 ^#^
median (IQR)	22 (20–24)	22 (20–24)	
mean (SD)	22.3 (3.1)	22.1 (3.3)	
**ASA score, *n* (%)**			1.000
I	295 (47.3)	295 (47.3)	
II	245 (39.3)	245 (39.3)	
III	84 (13.5)	84 (13.5)	
**Comorbidities, *n* (%)**			0.816
No	388 (62.2)	384 (61.5)	
Yes	236 (37.8)	240 (38.5)	
**Type of resection, *n* (%)**			0.955
Distal gastrectomy	293 (47)	294 (47.1)	
Total gastrectomy	331 (53)	330 (52.9)	
**Tumor location, *n* (%)**			0.731
Distal third	290 (46.5)	284 (45.5)	
Middle third	149 (23.9)	161 (25.8)	
Upper third	185 (29.6)	179 (28.7)	
**pTNM AJCC stage, 8th edition, *n* (%)**			0.860
IA	101 (16.2)	91 (14.6)	
IB	49 (7.9)	43 (6.9)	
IIA	67 (10.7)	69 (11.1)	
IIB	73 (11.7)	68 (10.9)	
IIIA	112 (17.9)	117 (18.8)	
IIIB	120 (19.2)	117 (18.8)	
IIIC	102 (16.3)	119 (19.1)	
**Histology, *n* (%)**			0.767
Differentiated	405 (64.9)	400 (64.1)	
Poorly differentiated/undifferentiated	219 (35.1)	224 (35.9)	

LG: laparoscopic gastrectomy; GO: open gastrectomy; *n* = number; ^#^ = Mann–Whitney U test; SD: standard deviation; IQR: interquartile range.

**Table 2:** Operating findings, postoperative complications, and pathology by surgical technique compared in the matched cohort.

	Matched Cohort		
	LG *n* = 624	OG *n* = 624	*p*-Value
**Total operative time (minutes)**			**<0.0001 ^#^**
Median (IQR)	180 (150–210)	240 (180–300)	
Mean (SD)	192.7 (72.6)	243.7 (86.5)	
**Conversion to open surgery, *n* (%)**	12 (1.9)	N/A	N/A
**N. harvested lymph nodes**			**<0.0001 ^#^**
Median (IQR)	32 (24–40)	28 (22–38)	
Mean (SD)	33.2 (12.6)	31.3 (14.1)	
**N. metastatic lymph nodes**			0.400 ^#^
Median (IQR)	3 (0–11)	3 (0–11)	
Mean (SD)	7 (9.1)	7.6 (10.8)	
**R factors**			**0.021**
R0	604 (96.8)	587 (94.1)	
R + (R1 − R2)	20 (3.2)	37 (5.9)	
**Neoadjuvant chemotherapy, *n* (%)**			0.493
Yes	20 (3.2)	15 (2.4)	
No	604 (96.8)	609 (97.6)	
**Neoadjuvant radiotherapy, *n* (%)**			1.000
Yes	1 (0.2)	0 (0)	
No	623 (99.8)	624 (100)	
**Histology types in detail, *n* (%)**			0.767
***Differentiated (total)***	*405 (64.9)*	*400 (64.1)*	
Tubular well-differentiated	358 (57.4)	301 (48.2)	
Tubular moderately differentiated	40 (6.4)	91 (14.6)	
Papillary	7 (1.1)	8 (1.3)	
***Poorly differentiated/Undifferentiated (total)***	*219 (35.1)*	*224 (35.9)*	
Mucinous	69 (11.1)	50 (8)	
Signet ring cell	97 (15.5)	96 (15.4)	
Poorly differentiated solid/non-solid type *	43 (6.9)	76 (12.2)	
Undifferentiated	10 (1.6)	2 (0.3)	
**Postoperative hospital stay (days)**			**<0.0001 ^#^**
Median (IQR)	10 (9–13)	14.8 (10–17)	
Mean (SD)	12.2 (8.2)	13 (7.6)	
**Patients with complications, *n* (%)**	94 (15.1)	133 (21.3)	**0.004**
**Clavien-Dindo complications, *n* (%)**			0.433
I	9 (6.4)	16 (9.3)	
II	106 (74.6)	121 (70)	
IIIa	8 (5.6)	7 (4)	
IIIb	8 (5.6)	17 (9.8)	
IVa	8 (5.6)	10 (5.8)	
IVb	2 (1.4)	0 (0)	
V	1 (0.8)	2 (1.1)	
Total	142 (100)	173 (100)	
**Severe in-hospital complications, *n* (%)**			0.689
Clavien-Dindo ≥ 3	27 (19)	36 (20.8)	
**Reoperation, *n* of patients (%)**	10 (1.6)	17 (2.7)	0.392
**In-hospital mortality, *n* (%)**	1 (0.2)	2 (0.3)	0.563

OG: open gastrectomy; LG: laparoscopic gastrectomy; *n* = number; # = Mann–Whitney U test; ml: milliliter; N/A: not applicable; IQR: interquartile range; SD: standard deviation; * only 2 patients with poorly differentiated: non-solid type (por2).

**Table 3:** In-hospital complications in the matched cohort.

	Matched Cohort		
	Laparoscopic *n* = 624	Open *n* = 624	*p*-Value
Acute pancreatitis	0	0	1.000
Acute renal failure	0	0	1.000
Adhesive ileus	0	0	1.000
Anastomotic stenosis	2	0	0.499
Anostomosis leakage	12	12	1.000
Arrhythmias	4	4	1.000
Atelectasia	1	0	1.000
Bleeding (intra/extraluminal)	9	10	1.000
Cholecystitis	0	0	1.000
Chylous leakage	6	8	0.789
Congestive heart failure	3	1	0.624
Cerebrovascular accident	0	0	1.000
Deep vein thrombosis	2	0	0.499
Delayed gastric emptying	8	7	1.000
Delirium	0	0	1.000
Disseminated intravascular coagul.	1	0	1.000
Dizziness	0	0	1.000
Dumping syndrome	0	0	1.000
Intra-abdominal fluid collection	14	9	0.400
Incisional hernia	1	2	1.000
Liver failure	0	0	1.000
Myocardial infarction	1	0	1.000
Omental infarction	0	1	1.000
Pancreatic fistula	2	6	0.287
Pleural effusion	3	2	1.000
Pneumonia	33	61	**0.003**
Prolonged postoperative ileus	1	1	1.000
Pseudomembranous colitis	2	2	1.000
Pulmonary edema	0	1	1.000
Pulmonary embolism	1	0	1.000
Remnant stomach necrosis	1	0	1.000
Sepsis	1	0	1.000
Small bowel infarction	0	2	0.499
Small bowel perforation	0	3	0.249
Unexplained postoperative fever	2	1	1.000
Wound infection	9	15	0.302
Wound seroma	0	1	1.000
Other complications	23	24	1.000
Total	142	173	**0.043**

*p*-Values in bold are statistically significant.

**Table 4:** Five-year cumulative overall survival (stratified by AJCC pTNM stage, 8th edition) in laparoscopic and open groups.

	*N* of Patients		Five-Year Survival Rate		*p*-Value *
	Laparoscopy	Open	Laparoscopy %	Open %	
**Entire cohort**					
All patients	1812	1221	81.1	66.4	<0.0001
Stage IA	358	199	97.4	93.5	0.029
Stage IB	156	130	94.2	75	<0.0001
Stage IIA	191	161	90.4	70.3	<0.0001
Stage IIB	213	144	88.5	79	0.002
Stage IIIA	309	231	78.5	56.6	<0.0001
Stage IIIB	352	195	69	51	<0.0001
Stage IIIC	233	161	60.1	48.5	0.001
**Matched cohort**					
All patients	624	624	77.4	75.2	0.202
Stage I	150	134	96.4	94	0.432
Stage IA	101	91	95	98	0.189
Stage IB	49	43	100	82	0.011
Stage II	140	137	86.7	83.8	0.469
Stage IIA	67	69	87	77.2	0.146
Stage IIB	73	68	86.6	91	0.509
Stage III	334	353	67.8	65	0.264
Stage IIIA	112	117	74.8	74.6	0.679
Stage IIIB	120	117	69	63.7	0.406
Stage IIIC	102	119	59.5	58.2	0.621

* Log-rank test.

**Table 5:** Univariate and multivariate Cox regression for five-year overall survival, entire cohort.

Overall Cohort		Univariable Cox Regression			Multivariable Cox Regression	
	*N*	Hazard Rate (95% CI)		*p*-Value	Hazard Rate (95% CI)	*p*-Value
**Age, years**	3033	1.029 (1.021–1.036)		**<0.0001**	1.019 (1.011–1.027)	**<0.0001**
**Gender**						
Male	2165	Reference				
Female	868	1.249 (1.050–1.485)		**0.012**		
**Geographic area**						
East	2187	Reference			Reference	
West	846	3.718 (3.137–4.406)		**<0.0001**	6.482 (5.322–7.895)	**<0.0001**
**Year of surgery**						
2000–2007	433	Reference				
2008–2014	2600	0.342 (0.281–0.415)		**<0.0001**		
**Body mass index (BMI)**	3033	1.027 (1.004–1.050)		**0.021**		
**ASA score**						
I	1508	Reference			Reference	
II	1211	1.674 (1.413–1.982)		**<0.0001**	1.273 (1.065–1.521)	**0.008**
III	314	2.387 (1.789–3.184)		**<0.0001**	1.338 (0.980–1.827)	0.067
**Operative time**	3033	1.002 (1.001–1.003)		**<0.0001**		
**Surgical approach**						
Open	1221	Reference				
Laparoscopic	1812	0.426 (0.363–0.500)		**<0.0001**		
**Length of hospital stay**	3033	1.010 (1.001–1.018)		**0.012**		
**Type of resection**						
Distal gastrectomy	1365	Reference				
Total gastrectomy	1668	2.011 (1.685–2.400)		**<0.0001**		
**Tumor location**						
Upper third	894	Reference			Reference	
Middle third	819	0.968 (0.798–1.174)		0.740	0.859 (0.707–1.045)	0.129
Distal third	1320	0.519 (0.427–0.632)		**<0.0001**	0.772 (0.633–0.941)	**0.011**
**N. harvested lymph nodes**	3033	1.009 (1.003–1.015)		**0.003**		
**Margin status**						
Free	2939	Reference				
Infiltrated	94	3.072 (2.192–4.306)		**<0.0001**		
**pTNM stage, 8th edition**						
IIIC	394	Reference			Reference	
IIIB	547	0.809 (0.651–1.006)		0.056	0.810 (0.604–1.805)	0.157
IIIA	540	0.657 (0.521–0.827)		**<0.0001**	0.645 (0.454–0.916)	**0.014**
IIB	357	0.305 (0.217–0.429)		**<0.0001**	0.282 (0.179–0.443)	**<0.0001**
IIA	352	0.345 (0.248–0.480)		**<0.0001**	0.261 (0.165–0.412)	**<0.0001**
IB	286	0.258 (0.173–0.386)		**<0.0001**	0.171 (0.102–0.288)	**<0.0001**
IA	557	0.073 (0.045–0.118)		**<0.0001**	0.057 (0.032–0.102)	**0.0004**
**N. of metastatic lymph nodes**	3111	1.040 (1.034–1.045)	0.039	**<0.0001**	1.018 (1.006–1.030)	**0.004**
**Histology, *n* (%)**						
Tubular, Well-diff. (tub1)	1596	Reference				
Tubular, mod. diff. (tub2)	368	3.331 (2.541–4.367)		**<0.0001**		
Papillary (pap)	51	1.535 (0.840–2.805)		0.163		
Mucinous (muc)	236	1.422 (1.062–1.905)		**0.018**		
Signet ring cell (sig)	484	1.801 (1.452–2.235)		**<0.0001**		
Poorly diff. solid/non-solid (por1/2) *						
Undifferentiated	275	4.573 (3.481–6.008)		**<0.0001**		
carcinoma	23	3.907 (1.933–7.897)		**<0.0001**		

*N*: number of patients; CI: confidence interval; B: beta coefficient; * poorly differentiated: non-solid type (por2) only 10 patients.

## Data Availability

Further information is available from the corresponding author on reasonable request.

## Supplementary Materials

The following are available online at https://www.mdpi.com/article/10.3390/cancers13184526/s1, Figure S1: Five-years overall survival, matched cohort in stage I (IA-IB) patients, Figure S2: Five-years overall survival, matched cohort in stage II (IIA-IIB) patients, Figure S3: Five-years overall survival, matched cohort in stage III (IIIA-IIIB-IIIC) patients, Figure S4: Five-years overall survival, matched cohort in Stage IA patients, Figure S5: Five-years overall survival, matched cohort in stage IIA patients, Figure S6: Five-years overall survival, matched cohort in stage IIB patients, Figure S7: Five-years overall survival, matched cohort in stage IIIA patients, Figure S8: Five-years overall survival, matched cohort in stage IIIB patients, Figure S9: Five-years overall survival, matched cohort in stage IIIC patients, Table S1: Clinical, surgical and demographic patients’ characteristics by surgical technique compared in the entire cohort, Table S2: Operating findings and postoperative complications and pathology by surgical technique compared in the entire cohort, Table S3: In hospital complications in the entire cohort.
