# Supplementary material for: Laparoscopic Compared with Open D2 Gastrectomy on Perioperative and Long-Term, Stage-Stratified Oncological Outcomes for Gastric Cancer: A Propensity Score-Matched Analysis of the IMIGASTRIC Database
Source: Cancers (Basel). 2021 Sep 8;13(18):4526. doi: 10.3390/cancers13184526 (PMC8465518; doi:10.3390/cancers13184526)
Supplement: Supplementary file 1 [file cancers-13-04526-s001.zip › cancers-1236485-supplementary.pdf]

Table S1. Clinical, surgical and demographic patients' characteristics by surgical technique compared in the entire cohort.

	Entire cohort		
	**Laparoscopic** *n* **= 1.812**	**Open** *n* **= 1.221**	***P* value**
**Age, years**			
median (IQR)	61(54-70)	66 (57-74)	0,097
mean (SD)	61,3 (11,6)	64,6 (11,9)	
**Gender, *n* (%)**			
Male	1.337 (73,8)	828 (67,8)	<0,0001
Female	475 (26,2)	393 (32,2)	
**Geographic area, *n* (%)**			
East	1.673 (92,3)	514 (42,1)	<0,0001
West	139 (7,7)	707 (57,9)	
**Year of surgery, *n* (%)**			
2000-2007	16 (0,9)	417 (34,2)	<0,0001
2008-2014	1.796 (99,1)	804 (65,8)	
**Body Mass Index (BMI),**			
median (IQR)	22 (20-24)	22,6 (21-25)	<0,0001
mean (SD)	22,3 (3,1)	23,1 (3,9)	
**ASA score, *n* (%)**			
I	1.054 (58,2)	454 (37,2)	<0,0001
II	625 (34,5)	586 (48)	
III	133 (7,3)	181 (14,8)	
**Comorbidities, *n* (%)**			
No	1.263 (69,7)	723 (59,2)	<0,0001
Yes	549 (30,3)	498 (40,8)	
**Type of resection, *n* (%)**			
Distal gastrectomy	840 (46,4)	525 (43)	0,068
Total gastrectomy	972 (53,6)	696 (57)	
**Tumor location, *n* (%)**			
Distal third	819 (45,2)	501 (41)	0,026
Middle third	460 (25,4)	359 (29,4)	
Upper third	533 (29,4)	361 (29,6)	
**pTNM AJCC stage 8th edition, *n* (%)**			
IA	358 (19,8)	199 (16,3)	0,005
IB	156 (8,6)	130 (10,6)	
IIA	191 (10,5)	161 (13,2)	
IIB	213 (11,8)	144 (11,8)	
IIIA	309 (17,1)	231 (18,9)	
IIIB	352 (19,4)	195 (16)	
IIIC	233 (12,9)	161 (13,2)	
**Histology, *n* (%)**			
Differentiated	1.352 (74,6)	663 (54,3)	<0,0001
Poorly differentiated/undifferentiated	460 (25,4)	558 (45,7)	

LG: Laparoscopic gastrectomy; GO: Open gastrectomy; *n* = number; SD: Standard deviation; IQR: Interquartile range.

Table S2. Operating findings and postoperative complications and pathology by surgical technique compared in the entire cohort.

	Entire cohort		
	**LG** *n*= 1.812	**OG** *n* =1.221	***P* value**
**Total operative time (minutes),**			
Median (IQR)	180 (150-199)	210 (180-270)	<0,0001[#]
Mean (SD)	182 (59)	227 (79,2)	
**Conversion to open surgery, *n* (%)**	12 (0,7)	N/A	N/A
**Type of reconstruction, *n* (%)**			
Billroth I gastroduodenostomy	642 (35,4)	143 (11,7)	< 0,0001
Billroth II gastrojejunostomy	115 (6,3)	131 (10,7)	
Jeiunal interposition	3 (0,2)	20 (1,6)	
Roux-en-Y esophagojejunostomy	970 (53,5)	676 (55,4)	
Roux-en-Y gastrojejunostomy	82 (4,5)	251 (20,6)	
**N. harvested lymphnodes**			
Median (IQR)	32 (25-41)	29 (22-38)	<0,0001[#]
Mean (SD)	33,8 (12,7)	31,4 (14)	
**N. metastatic lymphnodes**			
Median (IQR)	2 (0-9)	2 (0-8)	0,677[#]
Mean (SD)	6,1 (8,5)	6 (9,3)	
**R factors**			
R0	1.780 (98,2)	1.145 (93,8)	<0,0001
R+ (R1-R2)	32 (1,8)	76 (6,2)	
**Neoadjuvant chemotherapy, *n* (%)**			
Yes	47 (2,6)	77 (6,3)	<0,0001
No	1.765 (97,4)	1.144 (93,7)	
**Neoadjuvant radiotherapy, *n* (%)**			
Yes	1 (0,1)	6 (0,5)	0,038
No	1.811 (99,9)	1.215 (95,5)	
**Histology types in detail, *n* (%)**			
Differentiated (total)			
Tubular well-differentiated	*1.352* (74,6)	*663* (54,3)	<0,0001
Tubular moderately-differentiated	1.264 (69,8)	332 (27,2)	
Papillary	56 (3,1)	312 (25,6)	
Poorly differentiated/Undifferentiated	32 (1,8)	19 (1,6)	
(total)	*460 (25,4)*	*558 (45,7)*	
Mucinous	154 (8,5)	82 (6,7)	
Signet ring cell	239 (13,2)	245 (20,1)	
Poorly differentiated solid/non solid	55 (3)	220 (18)	
type*	12 (0,7)	11 (0,9)	
Undifferentiated			
**Postoperative hospital stay (days)**			
Median [IQR]	11 [9-13]	12 [10-15]	<0,0001[#]
Mean (SD)	12,4 (7)	14,4 (10,2)	
**Patients with complications, *n* (%)**	271 (15)	289 (23,7)	<0,0001
**Clavien-Dindo complications, *n* (%)**			
I	14 (3,7)	58 (15,1)	<0,0001
II	292 (80)	217 (56,6)	
IIIa	23 (6,3)	30 (7,8)	
IIIb	20 (5,4)	50 (13)	
IVa	12 (3,3)	13 (3,4)	
IVb	4 (1)	4 (1)	
V	1 (0,3)	12 (3,1)	
Total	366 (100)	384 (100)	
**Severe in hospital complications, *n* (%)**			<0,0001
Clavien-Dindo ≥3	60 (16,4)	109 (28,4)	

**Reoperation, n of patients (%)**	22 (1,2)	54 (4,4)	<0,0001
**In hospital mortality, *n* (%)**	1 (0,05)	12 (1)	<0,0001

OG: Open gastrectomy; LG: Laparoscopic gastrectomy; *n* = number; #= Mann-Whitney U test; ml: millilitri; N/A: Not applicable; IQR: Interquartile range; SD: Standard deviation; * Only 2 patients with Poorly differentiated: Non-solid type (por2).

Table S3. In hospital complications in the entire cohort.

	**Entire cohort**		
	**Laparoscopic** *n* **= 1.812**	**Open** *n* **= 1.221**	***P* value**
Acute Pancreatitis	0	1	0,842
Acute renal failure	0	2	0,316
Adhesive ileus	0	3	0,128
Anastomotic stenosis	2	2	1.000
Anostomosis leakage	32	44	**0,002**
Arrhythmias	8	9	0,411
Atelectasia	1	2	0,730
Bleeding (intra/extraluminal)	26	22	0,377
Cholecystitis	0	1	0,842
Chylous leakage	20	9	0,408
Congestive heart failure	3	1	0,910
Cerebrovascular accident	0	1	0,842
Deep vein thrombosis	2	0	0,659
Delayed gastric emptying	22	10	0,388
Delirium	0	1	0,842
Disseminated intravascular coagul.	2	0	0,659
Dizziness	0	1	0,842
Dumping syndrome	0	2	0,316
Intra-abdominal fluid collection	43	26	0,751
Incisional hernia	2	3	0,656
Liver failure	0	1	0,842
Myocardial infarction	1	2	0,730
Omental infarction	0	1	0,842
Pancreatic fistula	5	17	**0,0009**
Pleural effusion	3	8	0,058
Pneumonia	96	80	0,170
Prolonged postoperative ileus	1	4	0,174
Pseudomembranous colitis	2	3	0,656
Pulmonary edema	0	2	0,316
Pulmonary embolism	1	0	0,842
Remnant stomach necrosis	1	0	0,842
Sepsis	2	15	**0,0001**
Small bowel infarction	0	2	0,316
Small bowel perforation	0	4	0,053
Unexplained postoperative fever	2	3	0,656
Wound infection	30	25	0,512
Wound seroma	0	4	0,053
Other complications	59	73	**0,0004**
Total	366	384	**<0,0001**

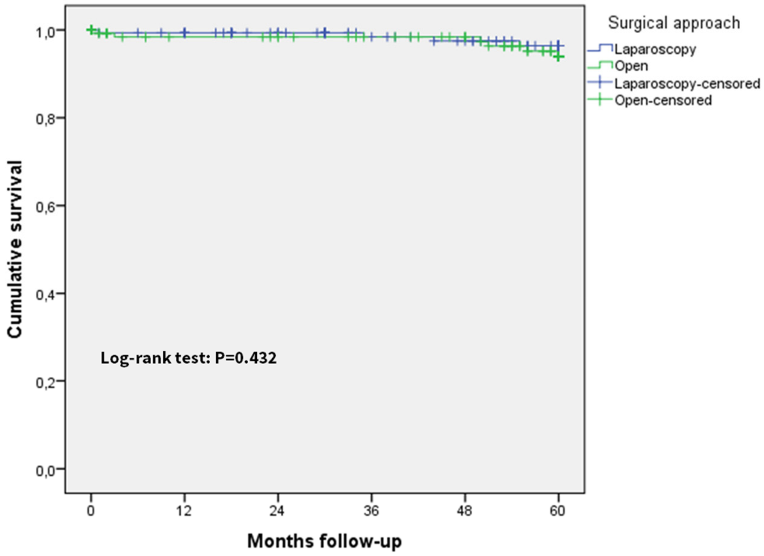

No. at Risk	Baseline	12 months	24 months	36 months	48 months	60 months
Laparoscopic	150	146	129	108	101	84
Open	134	111	109	103	98	75

Figure S1. Five-years overall survival, matched cohort in stage I (IA-IB) patients.

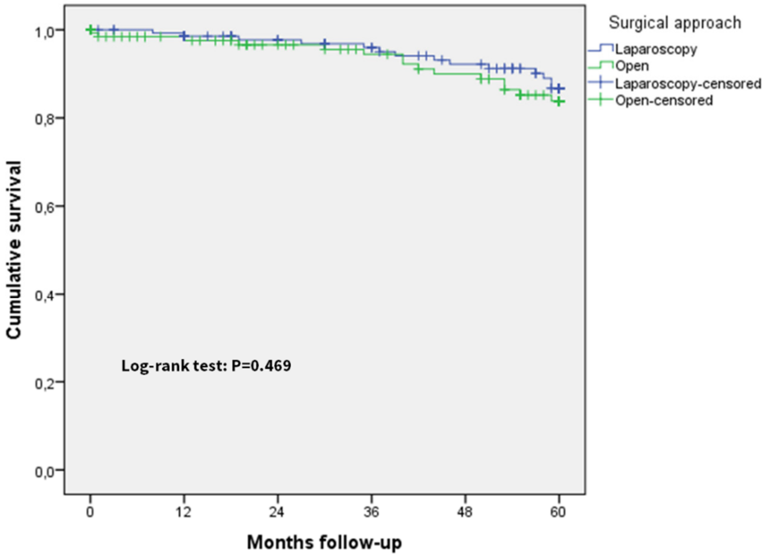

No. at Risk	Baseline	12 months	24 months	36 months	48 months	60 months
Laparoscopic	140	136	118	107	96	75
Open	137	110	95	86	79	61

Figure S2. Five-years overall survival, matched cohort in stage II (IIA-IIB) patients.

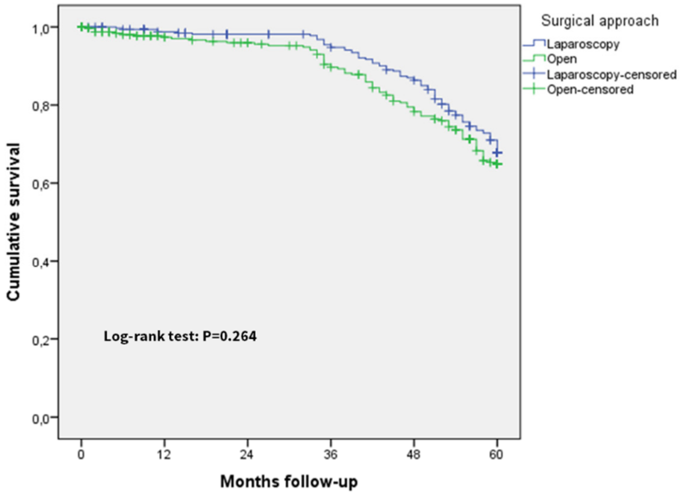

No. at Risk	Baseline	12 months	24 months	36 months	48 months	60 months
Laparoscopic	334	315	296	284	257	197
Open	353	278	266	243	208	150

Figure S3. Five-years overall survival, matched cohort in stage III (IIIA-IIIB-IIIC) patients.

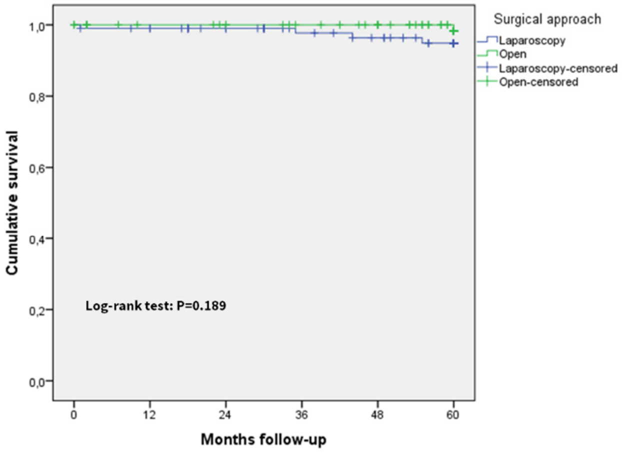

No. at Risk	Baseline	12 months	24 months	36 months	48 months	60 months
Laparoscopic	101	98	88	75	70	61
Open	91	84	82	77	73	57

Figure S4. Five-years overall survival, matched cohort in Stage IA patients.

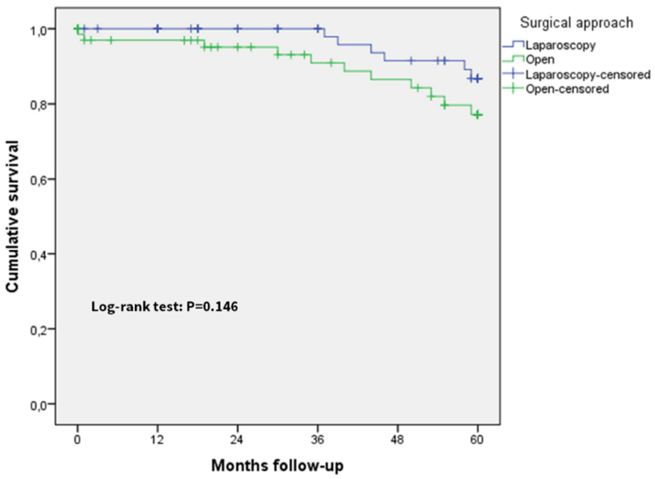

No. at Risk	Baseline	12 months	24 months	36 months	48 months	60 months
Laparoscopic	67	65	54	50	43	35
Open	69	57	49	42	39	31

Figure S5. Five-years overall survival, matched cohort in stage IIA patients.

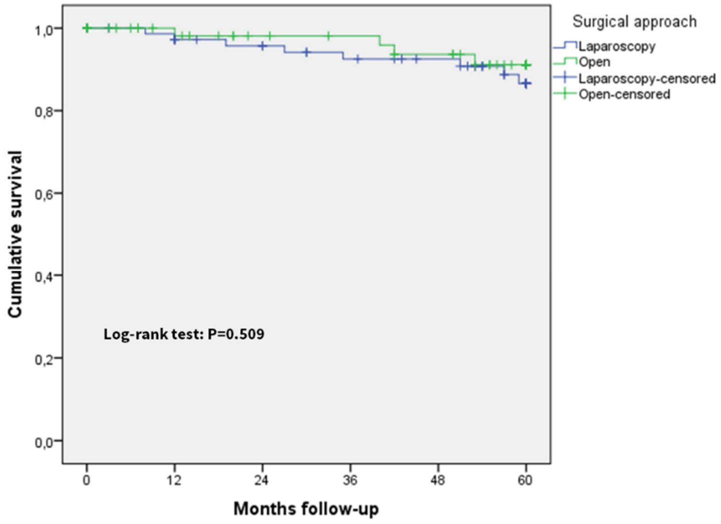

No. at Risk	Baseline	12 months	24 months	36 months	48 months	60 months
Laparoscopic	73	71	64	57	53	40
Open	68	53	46	44	40	30

Figure S6. Five-years overall survival, matched cohort in stage IIB patients.

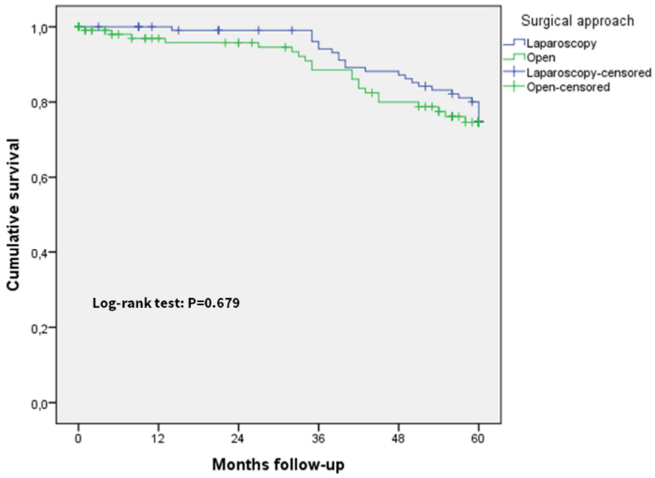

No. at Risk	Baseline	12 months	24 months	36 months	48 months	60 months
Laparoscopic	112	107	102	97	89	76
Open	117	86	83	73	65	47

Figure S7. Five-years overall survival, matched cohort in stage IIIA patients.

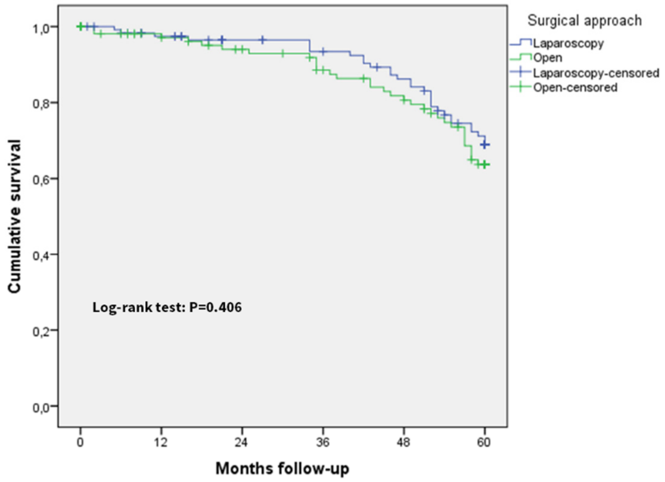

No. at Risk	Baseline	12 months	24 months	36 months	48 months	60 months
Laparoscopic	120	110	97	92	83	64
Open	117	97	89	80	72	50

Figure S8. Five-years overall survival, matched cohort in stage IIIB patients.

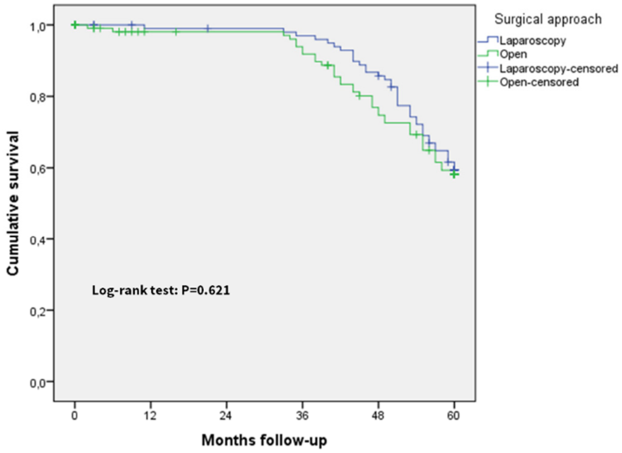

No. at Risk	Baseline	12 months	24 months	36 months	48 months	60 months
Laparoscopic	102	98	97	95	85	57
Open	119	95	94	90	71	53

Figure S9. Five-years overall survival, matched cohort in stage IIIC patients.
